# Spine surgery in a state-of-the-art hybrid operating room: an experience of 1745 implanted pedicle screws in the thoracolumbar spine

**DOI:** 10.1007/s11701-023-01533-x

**Published:** 2023-01-16

**Authors:** K. Schuetze, B. Rau, C. Dehner, M. Schultheiss, P. Richter, R. Cintean, F. Gebhard, A. Eickhoff

**Affiliations:** Department of Trauma-, Hand- and Reconstructive Surgery, Ulm University, Albert-Einstein-Allee 23, 89081 Ulm, Germany

**Keywords:** Spine surgery, Hybrid operating room, Pedicle screw accuracy, Thoracolumbar fractures

## Abstract

Hybrid-operating rooms (hybrid-OR) combine high-resolution 2D images and 3D-scans with the possibility of 3D-navigation and allow minimal invasive pedicle screw placement even in the upper thoracic spine. The disadvantage of high cost and increased radiation needs to be compensated with high accuracy and safety. The hybrid operating room consists of a floor-based flat-panel robotic C-arm with 3D-scan capability (Artis Zeego, Siemens; Germany) combined with navigation (BrainLAB Curve, BrainLAB; Germany). Through a minimally invasive incision, a Jamshidi needle was advanced through the pedicle and a K-wire was placed. If 2D image quality did not allow safe placement 3D-navigation was used to place the *K*-wire. Position was controlled through a 3D-Scan and corrected if necessary before screw placement. Postoperative CTs evaluated screw perforation grade with grade I when completely within the pedicle, II < 2 mm, III 2–4 mm, and IV > 4 mm outside the pedicle. Overall, 354 screws were placed in T1–T6, 746 in the lower thoracic spine T7–T12 and 645 in the L1-L5. Navigation was mainly used in upper thoracic spine cases (31 of 57). In 63 out of 326 cases K-wire was corrected after the 3D-Scan. Overall, 99.1% of the screws showed perforation less than 2 mm. Mean radiation was 13.3 ± 11.7 mSv and significantly higher in the upper thoracic spine and in navigated procedures. Despite higher costs and radiation, the hybrid-OR allows highest accuracy and therefore patient safety in minimal invasive pedicle screw placement in the thoracic and lumbar spine.

## Background/introduction

**Fig. 1:**
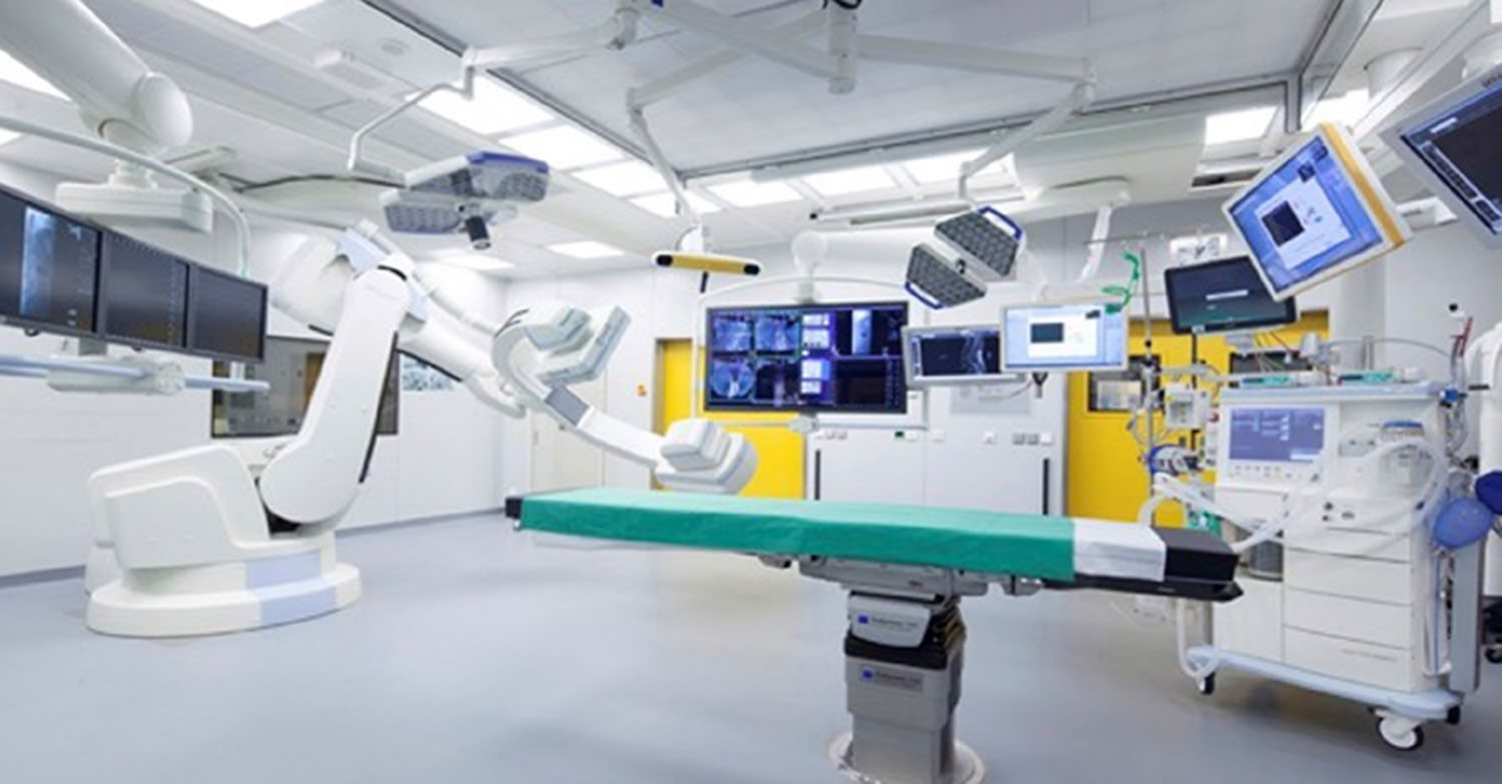
Hybrid-operating room

**Fig. 2:**
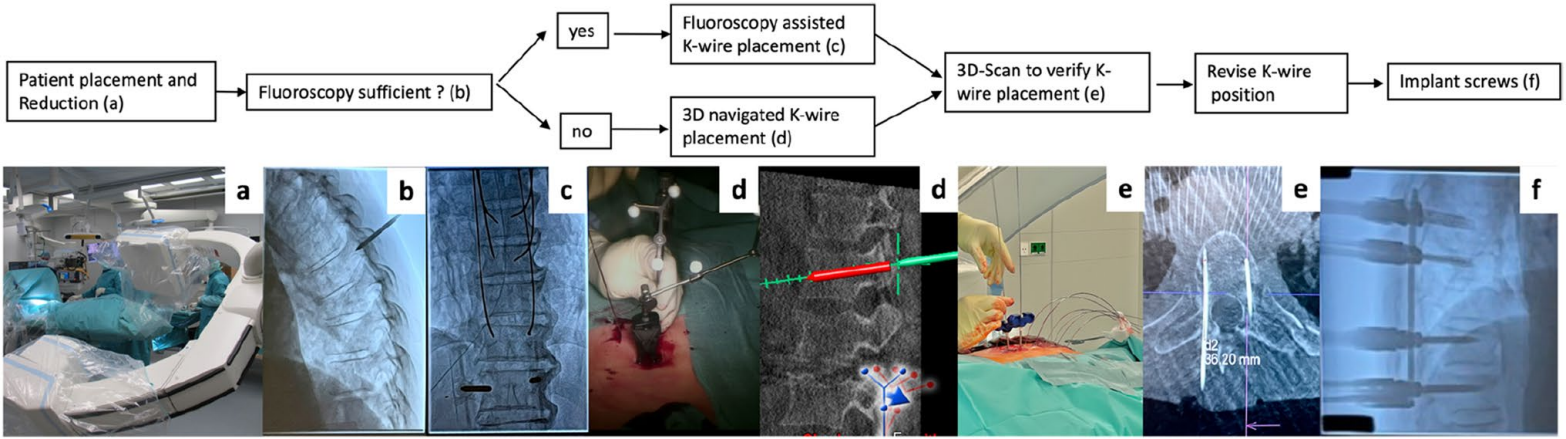
Algorithm for pedicle screw placement in the thoracolumbar spine

Hybrid-operating rooms are more and more commonly used in different fields of surgery like cardiothoracic surgery, vascular surgery, neurosurgery and orthopaedic trauma surgery especially at the spine and pelvis [1–5]. The improvement of visualization and intraoperative imaging, the 3D-Scan capability and possible computer assisted 3D navigation are clear advantages of a hybrid operating room which allows safe and minimal invasive surgery. Compared to a traditional operating room setting the imaging device is directly linked to operating table and navigation system and all devices are directly controlled by the attending surgeon. The combination of high-resolution images and easy to use 3D navigation if needed might allow the surgeon to increase the accuracy like in this study for pedicle screw placement even further. A recent systematic review and meta-analysis of 51.161 screws still found misplacement of less than 4 mm reaching from 5.0 to 18.9% depending on the used technique [6]. The authors of this study used since 2015 a newly build hybrid operating room for 5 years on a daily bases for all types of thoracolumbar fractures (Fig. 1). A spinal fracture algorithm was developed using fluoroscopy for minimal invasive pedicle screw placement under intraoperative control with 3D imaging and the possibility of 3D navigation if needed (Fig. 2). The aim of this study was to prove that an hybrid operating room can improve the accuracy of minimal invasive pedicle screw placement in fractures of the thoracolumbar spine.

## Methods

Institutional and prior ethical committee approval for the use of data in this study was obtained. Between June 2015 and December 2020, all patients with fractures of the spine were treated in newly build hybrid operating room (Fig. 1).

The special designed operating room had a room capacity of 55 square meters and consisted of a fixed robotic 3D flat-panel detector (Artis zeego, Siemens Healthineers, Germany), which is linked to the operating table (Trumpf, Germany) and a navigation system (BrainLab Curve, BrainLab, Germany). 326 patients with fractures of the thoracolumbar spine were included in the study. Figure 2 shows the developed and used algorithm of the authors. All included patients were treated in prone position and with a minimally invasive approach. Draping around the patient and for both branches allowed sterile free movement of the C-Arm (Fig. 2a). After initial fluoroscopy (Fig. 2b) if the pedicle was not clearly seen in anterior posterior (a.p.) views, a reference base was fixed to the processus spinosus near the fracture and intraoperative 3D scan was performed and automatically sent to the navigation system (Fig. 2d). In all cases, a Jamshidi needle was advanced under fluoroscopic control or with assistance of the navigation through the pedicle. After implanting a 1.45 mm guide wire (Fig. 2c) for all planned screws a 3D-Scan was performed with *K*-wires fixed flat using a sterile draping and a clamp. The 3D scan was then evaluated through the attending surgeon and the screw diameter and length were measured and planned (Fig. 2e). If one of the *K*-Wires was misplaced, it was corrected and the 3D-Scan was repeated. If all *K*-Wires showed perfect positioning the Screw-Rod-System (Viper2, DePuy Spine, 325 Paramount drive, Raynham, USA) was implanted (Fig. 2f).

For retrospective evaluation, clinical records including patient charts and radiographic images were reviewed. Patient-related factors like age, gender, body mass index and ASA classification were retrieved. Preoperative computed tomography images were used to classify the fractures applying the AO Spine thoracolumbar classification system [7]. Postoperative computed tomography images were used to assess screw placement. Screw perforation was divided in 4 grades according to Gertzbein et al. [8] (grade 0 perforation: no perforation, grade 1: 0–2 mm, grade 2: 2–4 mm, grade 3: > 4 mm).

The primary outcome measures were screw perforations and neurologic complications. The secondary outcome measures were used radiation and the number of changed *K*-wires after the 3D-Scan.

Data analysis was performed with IBM SPSS Statistics (V21.0) and Microsoft Excel (V16.3). Demographic characteristics are described as mean and standard deviation. For the primary outcome measures, ordinal regression was performed considering all variables related to the grade of screw perforation. For the secondary outcome measures, logistic regression was performed considering all variables related to the used radiation.

## Results

### Patient population

For 326 patients, the medical records were reviewed. Out of these 326 patients, 189 were male and 137 were woman. The mean age was 59.3 ± 20.1 years. Overall, there were 89 thoracic fractures between T1 and T10, 181 thoracolumbar fractures between T11 and L2 and 56 lumbar fractures between L3 and L5. All fractures were classified with the “AOSpine Thoracolumbar Classification System”. The fracture classification is shown in Table 1.

**Table 1:** Classification, number and diameter of screws and rate of K-wire correction in the different anatomical regions

	Thoracic fracture	Thoracolumbar fracture	Lumbar fracture
AO-Spine classification			
A1	3	10	2
A2	2	4	3
A3	50	130	41
A4	1	6	5
B1	4	5	1
B2	12	8	1
B3	11	11	2
C	6	7	1
Number of screws			
4	39	133	53
6	2	4	0
8	42	38	3
10	2	3	0
11	0	1	0
12	4	2	0
Diameter of screws			
4 mm	17	0	1
5 mm	47	25	1
6 mm	20	99	18
7 mm	5	56	34
8 mm	0	1	2
K-wire correction			
Yes	28	26	9
No	61	155	47

### K-Wire correction

After placing the *K*-wires and performing the 3D-Scan 63 out of 326 *K*-wires needed correction like shown in Table 1. Correction was needed more often in the thoracic spine (28 out of 89 cases) than in thoracolumbar spine (26 out of 181 cases) and lumbar spine (9 out of 56 cases). Significantly more *K*-wires were changed after navigated surgery. 18 out of 55 navigated cases needed K-wire correction while correction was only performed in 45 out of 271 non-navigated cases (32.7% vs 16.6%; *p* < 0.05). K-wire correction is shown in Table 2.

**Table 2:** Comparison of navigated and non-navigated procedures

	Navigated (%)	Non-navigated (%)
Region		
Thoracic	54.3	45.7
Thoracolumbar	9.8	90.2
Lumbar	8.2	91.8
Accuracy		
Grade 0	81.2	91.6
Grade I	15.3	8.1
Grade II	3.5	0.3
Grade III	0	0
K-wire correction	32.7	16.6

### Pedicle screw accuracy

**Fig. 3:**
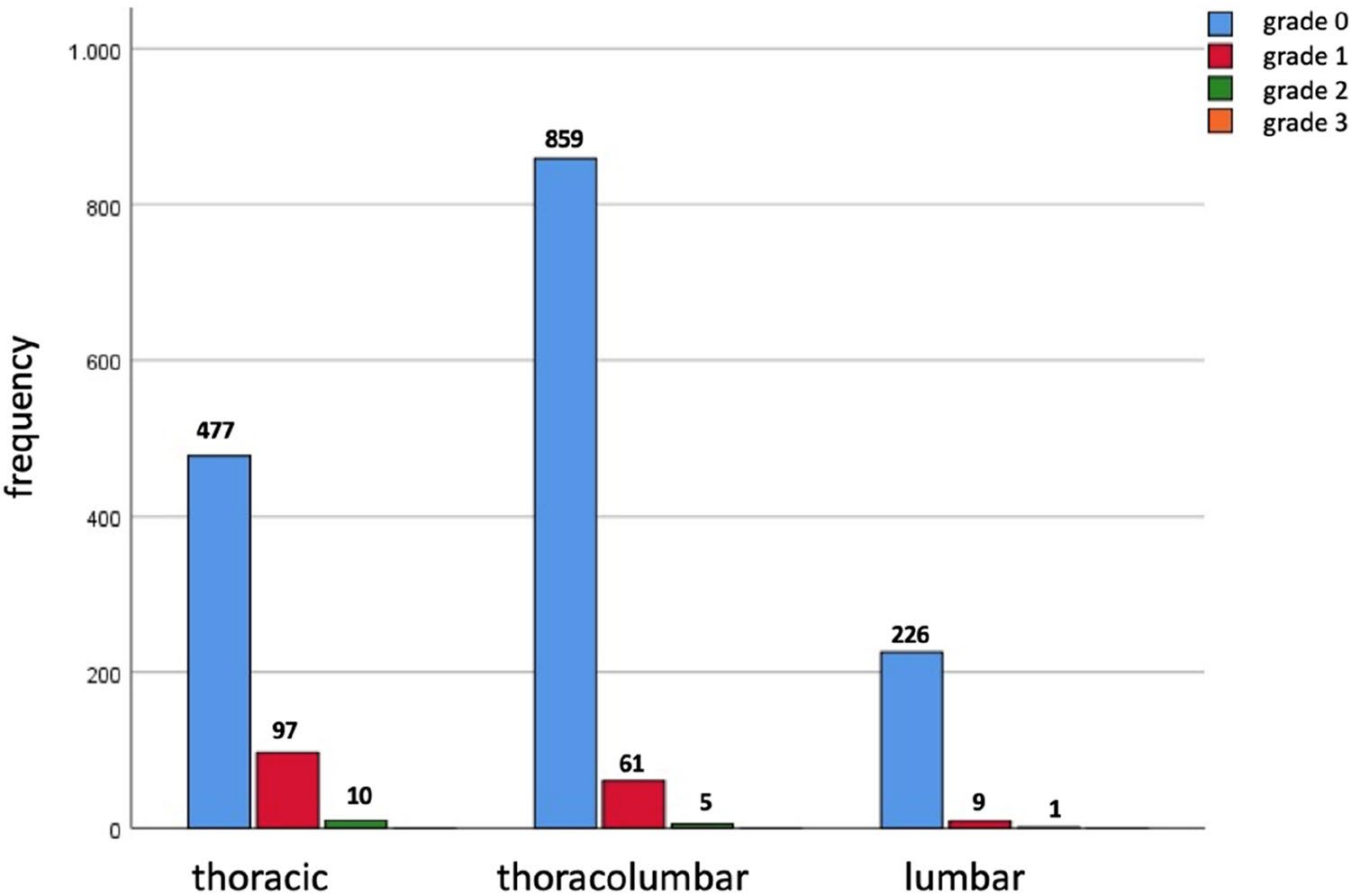
Screw perforation according to Gertzbein et al. (grade 0: no perforation, grade 1: 0–2 mm, grade 2: 2–4 mm, grade 3: > 4 mm)

1562 out of the 1745 implanted pedicle screws were completely inside the pedicle, 167 were classified as perforation grade I, 16 as grade III and 0 as grade IV. Thus 99.1% of the screws showed perforation less than 2 mm. While in the thoracic spine only 81.7% of the screws were completely inside the pedicle, there were 92.9% in the thoracolumbar and 95.8% in the lumbar spine. This difference between the groups was significant (*p* < 0.001). In terms of grade III perforations 10 were in the thoracic spine, 5 in the thoracolumbar and 1 in the lumbar spine. Most commonly screw diameter was 5 mm in the thoracic spine, 6 mm in the thoracolumbar spine and 7 mm in the lumbar spine. Between 4 and 16 screws were implanted with 4 screws being the most common amount of screws used. Number of screws and diameter are shown in Table 1. Perforations for each anatomical region of the spine are shown in Fig. 3.

### Navigated procedures

In only 16.9% of the patient’s intraoperative computer navigation was used for K-Wire placement. Navigation was used in 31 out of 57 upper thoracic spine cases, in 12 out of 123 thoracolumbar spine cases and in 12 out of 146 lumbar spine cases. In navigated procedures, there were significantly more grade I and grade II perforation as well as less screws completely placed in the pedicle (*p* < 0.05). A comparison between navigated and non-navigated procedures is shown in Table 2.

### Radiation dose and operating time

Mean radiation dose was 13.3 ± 11.7 mSv. It was significantly higher in the upper thoracic spine compared to the lumbar spine (thoracic 16.0 ± 11.6 mSv vs thoracolumbar 12.5 ± 11.4 mSv vs lumbar 11.3 ± 9.9 mSv; *p* < 0.05). Navigated surgery emitted significantly more radiation than non-navigated surgery (navigated 20.3 ± 14.1 mSv vs non-navigated 11.6 ± 10.6 mSv; *p* < 0.001). Operating time showed no significant difference between the 3 locations (thoracic 109 ± 63 min vs thoracolumbar 89 ± 65 vs lumbar 88 ± 53; *p* > 0.05). Navigated surgery showed a significantly longer operating time than non-navigated surgery (*p* < 0.05). Detailed operating times are shown in Table 3.

**Table 3:** Operating time for all 3 regions

	Thoracic (min)	Thoracolumbar (min)	Lumbar (min)
All cases	109 ± 63	89 ± 65	88 ± 53
Non-navigated	99 ± 65	86 ± 64	77 ± 38
Navigated	128 ± 52	140 ± 51	146 ± 85
Cases with 4 screws	89	68	56

## Discussion

This study showed that utilizing all the features of a hybrid operating room can improve the accuracy of pedicle screw placement for fractures on all thoracolumbar levels from T1 to L5. Compared to the systematic review of Perdomo-Pantoja et al. that reported an accuracy rate of 95.5% with CT-navigation and 91.5% in fluoroscopy assisted technique, we achieved a considerably lower screw perforation rate. Using the described algorithm in Fig. 2, perforation of more than 2 mm only occurred in 0.9% of the cases. This is one of the lowest evaluated rates of perforation compared to literature [6, 9]. After navigated or non-navigated *K*-Wire placement, a 3D-Scan was performed and misplaced *K*-Wires were corrected. This was necessary in 19.3% of the cases and significantly more often in navigated procedures. The replacement in these cases prevented perforation rates like seen in comparable studies. The higher rate on perforation in navigated procedures as well as the higher rate of replaced *K*-wire might be explained by smaller pedicles and more complex trajectories. Even with 3D navigation there is always a small percentage of misplacement. Compared to the study of Tjasic et al., who also used 3D-navigation, our study had more minor breaches [10]. While Tjasic et al. reported 1.6% perforation over 4 mm, only perforation under 4 mm were evaluated in this study which also can be explained by the K-wire correction. Liu et al. compared pedicle screw placement for fractures in the thoracic spine either using fluoroscopy or 3D-Scan via O-arm to control *K*-wire position. A significantly higher rate of satisfactory positioned pedicle screws (grade 0 and I) was observed in the O-arm control group [11]. The rate of perforations with less than 2 mm was with 96.6% comparable to the 98.3% in our study underlining the importance of a *K*-wire control after fluoroscopy guided pedicle screw placement. Perforation over 4 mm could be prevented in both studies. The slightly higher accuracy in our study might be explained by the use of 3D-navigation in complex cases with insufficient imaging. Comparable results for perforations less than 2 mm were seen by Shin et al. with 97.1% in the navigated group using O-arm navigation and 94.1% in the non-navigated group [12]. Still in both groups 1 vs 4 pedicle screws with perforations over 4 mm were found. Due to the K-wire correction after placement in both groups we could achieve a slightly higher accuracy and prevent perforation over 4 mm. In 16.9% of the cases 3D-navigation was used mainly in the thoracic spine because of insufficient fluoroscopy. In our study, navigated procedures showed 81.2% of the screws completely inside the pedicle and 15.3% showed perforation less than 2 mm. In comparison, Scarone et al. showed a higher rate of accuracy for O-arm and iCT-Airo navigated pedicle screw placement with 92.1% [13]. The rate of pedicle screws completely inside the pedicle also was higher in the study of Shin et al. with 93.3% and in the study of Tjasic et al. with 92.8% for 3D-C-Arm navigated and 98.9% for O-Arm navigated [12, 14]. The higher rates might be explained by the selection bias of our study that was not concepted as a comparison between navigated and non-navigated cases. Therefore the majority of navigated case were thoracic cases from T1-T10 with often poor imaging quality and small pedicle diameters, while Scarone et al. and Tajsic et al. placed more navigated screws in the lumbar spine [13, 14].

Main disadvantages of a hybrid operating room are extensive costs for the initial outlay of about 1.2 million dollars and the anticipated high radiation dose. While the costs for the initial outlay are high the operating time might be lower due to better imaging quality and through direct control of the C-Arm by the surgeon. Bronsard et al. reported an operating time of 83.5 min for mainly lumbar fractures with 4 percutaneously implanted screws using only fluoroscopy [15]. In comparison, mean operating time in the present study for 4 lumbar screws was 56 min. For fluoroscopic thoracic pedicle screw placement mean time was 99 min which is considerably faster than the study of Liu et al. with an average of 195 min. Navigated surgery showed significantly longer operating time which is in line with literature [9, 16]. Shin et al. demonstrated placing the screws with help of navigation is comparable time consuming than under fluoroscopic control but uses much more time for preparation [12]what might explain the longer operating time. Compared to O-Arm navigation with about 200 min operating time in different studies [9, 13] the operating time with 3D-C-Arm with navigation in this study was noticeable shorter with a mean time of 135 min. Also, with a comparable technique Tjasic et al. needed about 200 min. As described by Ryang et al. navigated procedures come with a steep learning curve and constant improvement for in radiation and operating time [17]. The authors are using 3D navigation since 2015 for pelvic and spinal surgeries and accumulated a lot of experience and cases what might explain the faster operating times.

As expected, thoracic cases needed more radiation dosage than thoracolumbar and lumbar cases. Furthermore, navigated cases used more radiation than non-navigated cases. Schuetze et al. showed that navigation reduces the radiation exposure for the operating personnel because it can leave the room during 3D-Scans [18, 19]. Furthermore, with the help of the ALARA principles radiation can be further reduced. The study of Bronsard et al. and Tajsic et al. had an effective dose of about 1.5 mSv only using fluoroscopy [14, 15]. Mean dosage for non-navigated cases was 11.6 mSv considerably higher but can be explained by the control 3D-Scan to verify *K*-Wire position. Furthermore, the 3D-Scan was even repeated if a *K*-Wire needed revision. Compared to the mentioned studies the accuracy of the pedicle screws was as mentioned above higher which in our opinion justifies the higher radiation dosage. For navigated procedures we measure a mean radiation dosage of 20.3 mSv. This is in line with a comparable study using iCT-Airo and O-Arm navigation [13].

## Conclusion

This study is the first study to evaluate the daily use of a state-of-the-art hybrid operating room in the treatment of spinal fractures from T1 to L5. Due to the high-resolution imaging, 3D-Scan controlled K-wire placement and 3D-navigation when fluoroscopy was not sufficient an accuracy of 99% for perforations less than 2 mm could be achieved. Still, costs for the initial outlay and radiation must be taken into account as a disadvantage.

## Data Availability

The data that support the findings of this study are available from the corresponding author upon reasonable request.
